# Gentamicin fails to eradicate *Staphylococcus aureus* biofilm in vitro, even in combination with rifampin

**DOI:** 10.5194/jbji-11-65-2026

**Published:** 2026-02-02

**Authors:** Willemijn Boot, Michel Schläppi, Virginia Post, T. Fintan Moriarty, Peter Wahl

**Affiliations:** 1 AO Research Institute Davos, 7270 Davos, Switzerland; 2 Division of Orthopaedics and Traumatology, Cantonal Hospital Winterthur, 8401 Winterthur, Switzerland; 3 Endo-Team, Birshof Hospital, 4142 Muenchenstein, Switzerland; 4 Department of Biomedical Engineering, University of Basel, 4123 Allschwil, Switzerland; 5 ARTORG Centre for Biomedical Engineering Research, Faculty of Medicine, University of Bern, 3008 Bern, Switzerland

## Abstract

**Introduction**: Biofilm formation is one of the key elements making orthopaedic device-related infections (ODRIs) difficult to eradicate. Aminoglycosides such as gentamicin are frequently applied via local carriers, and systemic rifampin is added for its anti-biofilm activity. However, robust in vitro evidence of their ability to eradicate mature biofilm is limited. This study assessed whether gentamicin, alone or in combination with rifampin, can eradicate established *Staphylococcus aureus* biofilm in vitro. **Methods**: A clinical methicillin-susceptible *S. aureus* isolate was grown as a 5 d old biofilm on a peg lid microtiter plate. Three exposure regimens were tested: (i) continuous exposure to gentamicin (15–2000 mg L^−1^) for 28 d, (ii) intermittent 2 h exposures twice daily (at 15, 250 and 2000 mg L^−1^) for 28 d to reflect systemic twice-daily dosing and (iii) a 14 d burst release starting at 2000 mg L^−1^ with stepwise decline to model release from local carriers. Rifampin was either absent or added at 3.3 mg L^−1^, approximating peri-implant concentrations from preclinical pharmacokinetic studies. Biofilm viability was quantified as colony-forming units (CFUs) from sonicated pegs, and selected surviving isolates underwent susceptibility testing. **Results**: Across all regimens, concentration- and time-dependent decreases in CFU counts were observed, but no regimen resulted in bacterial counts falling below the lower limit of detection (LLOD). The addition of rifampin did not result in the sustained enhancement of biofilm killing, and, in some regimens, resulted in higher CFU counts. Isolates recovered from culture-positive pegs remained largely susceptible to gentamicin, whereas rifampin resistance arose sporadically. **Conclusion**: High-dose gentamicin exposures failed to eradicate 5 d old *S. aureus* biofilm in vitro, whatever the administration regimen. Rifampin co-administration did not alter the final outcome of biofilm persistence, despite its well-recognised anti-biofilm activity. These findings challenge the reliance on aminoglycoside-loaded carriers as curative strategies for ODRIs and suggest that persistent viability may reflect antibiotic tolerance that may not be overcome by antibiotics alone.

## 1 Introduction

Orthopaedic device-related infections (ODRIs) are notoriously difficult to treat due to multiple factors. Leucocyte dysfunction in close vicinity to implants (Zimmerli et al., 1984), bacterial seclusion in immune-privileged niches (Moriarty et al., 2021; Jensen et al., 2023; de Mesy Bentley et al., 2018; Hartmann et al., 2025), the development of phenotypic variants such as (intracellular) persisters and small-colony variants (Tuchscherr et al., 2010; Kalinka et al., 2014; Sendi and Proctor, 2009; Harms et al., 2016; Hamad et al., 2022), biofilm formation (Gristina, 1987; Costerton et al., 1987; Bjarnsholt et al., 2013b; Harms et al., 2016; Hartmann et al., 2025; Olsen, 2015; Høiby et al., 2010), and the development of antibiotic tolerance and resistance (Brauner et al., 2016; Olsen, 2015; Høiby et al., 2010; Choi et al., 2008) all contribute to the persistence of infection and treatment failure (Harms et al., 2016). Biofilm develops and matures within days and confers marked tolerance to environmental stresses, such as the action of the immune system and antibiotic drugs (Høiby et al., 2010; Stewart, 2015; Gristina, 1987; Bjarnsholt et al., 2013b; Costerton et al., 1987; Hartmann et al., 2025; Olsen, 2015).

The treatment of biofilm-associated infections requires months of biofilm-active systemic therapy (Bernard et al., 2021; Kusejko et al., 2021; Achermann et al., 2013). However, systemic antibiotic therapy is constrained by side effects, adverse reactions and toxicity issues (Valour et al., 2014). Poor penetration at the site of infection further limits efficacy (Mouton et al., 2008), whereas cell toxicity issues limit local delivery (Rathbone et al., 2011; Wiesli et al., 2021). Nevertheless, even mature biofilm may be cleared by antibiotics alone, at least under certain in vitro conditions, with sufficiently high concentration and exposure over approximately 1 month (Post et al., 2017; Baeza et al., 2019). Determining the necessary time-concentration thresholds is crucial for the optimisation of ODRI treatment, particularly via local delivery strategies (Reinisch et al., 2022; Wahl et al., 2017; Li et al., 2018; Tande and Patel, 2014; Metsemakers et al., 2020; Wiesli et al., 2022; Gramlich et al., 2020).

Aminoglycosides such as gentamicin and tobramycin remain among the antibacterial drugs most commonly applied locally in orthopaedic surgery (Metsemakers et al., 2020; Iarikov et al., 2012; Krause et al., 2016; Ferguson et al., 2023). The essential pharmacodynamic parameter determining bacterial killing of aminoglycosides is concentration (Turnidge, 1998; Krause et al., 2016). While aminoglycosides are used rarely for the systemic therapy of ODRI, their spectrum of activity, stability and concentration-dependent activity makes them attractive for local application, beyond historical considerations. Rifampin is frequently administered systemically additionally to other local or systemic agents in the treatment of ODRI for its anti-biofilm activity, but its synergy with aminoglycosides in this context remains unclear (Renz et al., 2021; Beldman et al., 2021). This study aimed to determine the in vitro time-concentration profiles required for the eradication of matured *Staphylococcus aureus* biofilm with gentamicin, alone or in combination with rifampin, using continuous, intermittent and burst-release regimens.

## 2 Methods

### 2.1 Biofilm formation

A multi-sensitive clinical *S. aureus* isolate, available at the Swiss Culture Collection (JAR 060131, Waedenswil, Switzerland; access number CCOS 890), was used for all experiments. The strain has a minimal inhibitory concentration (MIC) to gentamicin and to rifampin of ≤0.5 mg L^−1^. A stationary-phase culture tryptic soy broth (TSB, Oxoid, Pratteln, Switzerland) was used for the inoculum preparation. The 20 mL bacterial culture was centrifuged, washed with sterile room-temperature phosphate-buffered saline (PBS, Sigma-Aldrich, Buchs, Switzerland), sonicated for 3 min in an ultrasound water bath (Sonorex Super 10P, Bandelin, Berlin, Germany) operating at a frequency of 40 kHz and resuspended in 20 mL PBS. The bacterial suspension was adjusted to an optical density ranging between 1.1 and 1.2 (approximately 4–5 × 10^8^ colony-forming units (CFUs) mL^−1^), measured at 600 nm (OD_600_) using a spectrophotometer (Multiskan Go, Thermo Scientific, Zürich, Switzerland). The bacteria were then diluted 1000× in TSB with the addition of 1 % pooled human plasma (Reginal Blood Donation Service SRK Graubünden, Chur, Switzerland) for a final concentration of ∼ 10^5^ CFU mL^−1^.

The biofilm model used in the present study was the MBEC device (MBEC Assay Biofilm Inoculator with trough base, product code 19121, Innovotech, Edmonton, Alberta), described elsewhere (Ceri et al., 1999). Briefly, the MBEC device consists of a lid with 96 polystyrene pegs fitting over a trough base plate. For inoculation, 22 mL of the bacterial suspension were added to the trough base plate. Biofilms were grown on the pegs for 5 d, with agitation (30 rpm) at 37 °C. The culture medium was exchanged daily.

To verify biofilm formation, scanning electron microscopy (SEM) was performed on pegs removed daily until day 5. For this, pegs with biofilm were dehydrated through a series of ethanol steps (50 %, 60 %, 70 %, 80 %, 90 %, 96 % and 100 % ethanol for 5 min each) and then coated with 10 nm gold/palladium. Images were taken in the secondary electron mode with an accelerating voltage of 3 kV and an emission current of 40 µ A using an S4700 scanning electron microscope (Hitachi, Tokyo, Japan).

### 2.2 Antibiotic challenges

Antibiotic challenges were performed in microtiter plates (Nunclon Delta, Thermo Scientific, Reinach, Switzerland). After 5 d of biofilm formation, the peg lids were placed onto the challenge plates. Each well was filled with 200 µ L of a solution containing either TSB supplemented with 1 % human plasma without antibiotics for the control group or with gentamicin (Roth, Arlesheim, Switzerland) or with gentamicin and rifampin (Labatec Pharma, Meyrin, Switzerland) in combination. The following exposure regimens were tested, all without rifampin or with concomitant continuous rifampin concentrations of 3.3 mg L^−1^:

- continuous exposure to gentamicin at constant concentrations ranging from 15 to 2000 mg L^−1^ for 28 d;
- intermittent exposure to gentamicin for 2 h twice daily at concentrations of 15, 250 and 2000 mg L^−1^ for 28 d, with pegs kept in an antibiotic-free medium between exposure;
- burst release with exposure to gentamicin at degressive concentrations, starting at 2000 mg L^−1^ at day 0, reduced stepwise to 2 mg L^−1^ at day 14, as illustrated in Table 1.

Each condition used 18 pegs ( n=18 ) distributed across three independent biological replicates (six pegs per replicate), unless otherwise stated. For all exposure regimens, the challenge medium was refreshed every other day throughout the experiment, except for the intermittent exposure, where it was refreshed after the 2 h of exposure.

### 2.3 Quantitative microbiology

For quantification of the number of CFUs per peg, the pegs were broken off, placed in 10 mL Dey–Engley (DE) neutralising broth (D3435, Sigma-Aldrich) in glass vials and sonicated as described above for 30 min. The disrupted biofilms were diluted serially in PBS. Standard plating consisted of 100 µ L per plate onto tryptic soy agar (TSA, Oxoid) and incubation at 37 °C. When no colonies were recovered at this lowest dilution, 1 mL of the 10 mL sonicate was plated on a single agar plate to improve detection sensitivity. Accordingly, the effective lower limit of detection (LLOD) was 10 CFU per peg.

### 2.4 Antimicrobial sensitivity testing

Certain colonies from culture-positive pegs were sampled for susceptibility to gentamicin and to rifampin. An inoculum equivalent to 0.5 McFarland (corresponding to 1– $2\times 10^{8}$ CFU mL^−1^, corresponding to an OD_600_ of 0.08–0.09) was prepared by suspending colonies from the bacteria grown overnight in PBS. The solution was sonicated for 3 min, and the OD_600_ was adjusted to a range between 0.08 and 0.09 with PBS if needed. This suspension was used to inoculate Mueller–Hinton agar (MHA, Oxoid) plates. Thereafter, gentamicin (10 µ g, BD BBL Sensi Disc, Switzerland) or rifampin (5 µ g, Oxoid) discs were placed onto the agar. The diameter of the zone of inhibition was measured after 24 h of incubation of the plates at 37 °C under aerobic conditions. For data interpretation, results were compared with EUCAST zone diameter breakpoints (sensitive ≥18 mm, respectively resistant <18 mm for gentamicin and sensitive ≥26 mm; respectively resistant <26 mm for rifampin) (EUCAST, 2023).

**Table 1:** Concentrations of gentamicin used for the experiments simulating burst release from a local carrier.

Day of challenge	Concentration (mg L^−1^)
Day 0	2000
Day 1	200
Day 2	100
Day 3	80
Day 4	40
Day 5	20
Day 6	20
Day 7	10
Day 8	10
Day 9	5
Day 10	5
Day 11	2
Day 12	2
Day 13	2
Day 14	2

### 2.5 Statistical workup

Data descriptions and statistical analyses were performed depending on data characteristics. Scalar data are described by median and range when normal distribution was not ensured. Mean and standard deviation were used for normally distributed data. Normality was assessed visually using Q-Q plots and Tukey–Anscombe plots. For group comparison, linear mixed-effects models were fitted using lmer function from the lme4 and lmerTest packages in R (v4.4.2) to account for repeated measures. Categorical data were described using counts and proportions, and compared using the chi-squared test and Fisher's exact test, as appropriate. Statistical significance was accepted for p<0.05 .

## 3 Results

**Figure 1:**
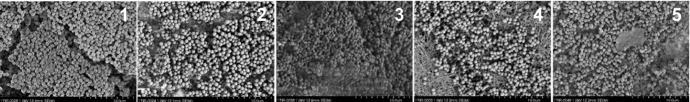
Scanning electron microscopy images ( 5000× ) of the *S. aureus* biofilm formation during the 5 d of growth and maturation on the polystyrene pegs. The numbers indicate the number of days of the sample.

Biofilm formed and matured as desired, documented by the daily SEM verification (Fig. 1), confirming dense surface coverage and three-dimensional matrix development by day 5.

**Figure 2:**
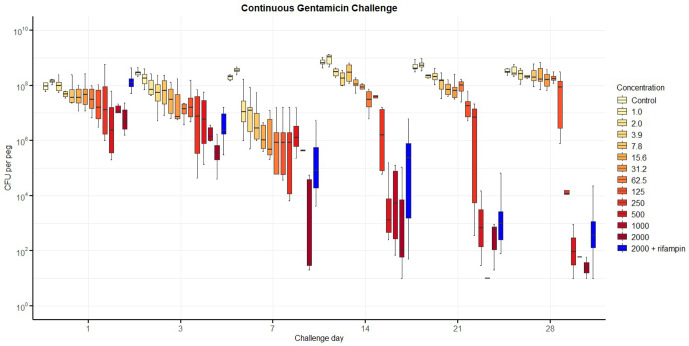
Number of colony-forming units (CFUs) recovered from biofilm over time after continuous exposure to gentamicin, including with additional rifampin. The horizontal bar indicates the median value, the box is the interquartile range (IQR) and the whiskers represent values within 1.5× IQR of the lower and upper quartiles. None of the regimens allowed the complete elimination of viable bacteria. The addition of rifampin (3.3 mg L^−1^) to gentamicin at 2000 mg L^−1^ performed less well than gentamicin alone.

**Figure 3:**
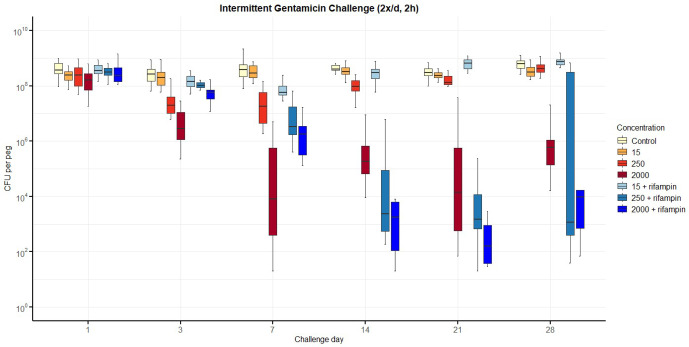
Number of colony-forming units (CFUs) over time for intermittent exposure to gentamicin twice daily, including with additional rifampin. The horizontal bar indicates the median value, the box is the interquartile range (IQR) and the whiskers represent values within 1.5× IQR of the lower and upper quartiles. None of the regimens resulted in bacterial counts falling below the lower limit of detection (LLOD), even if additional rifampin (3.3 mg L^−1^) improved bacterial reduction for gentamicin at 250 and 2000 mg L^−1^.

**Figure 4:**
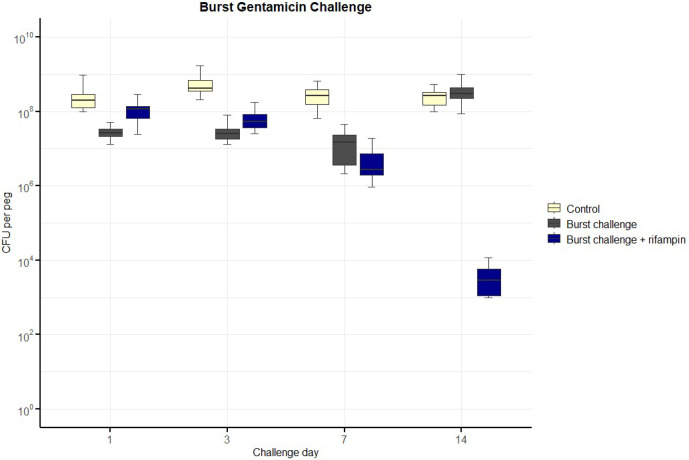
Number of colony-forming units (CFUs) over time for exposure to a burst release of gentamicin, including with additional rifampin. The horizontal bar indicates the median value, the box is the interquartile range (IQR) and the whiskers represent values within 1.5× IQR of the lower and upper quartiles. None of the regimens resulted in bacterial counts falling below the lower limit of detection (LLOD). The concentrations of gentamicin over time are indicated in Table 1. Rifampin was added at 3.3 mg L^−1^.

Results from the three gentamicin exposure regiments are summarised in Figs. 2 to 4.

For *continuous exposure* (Fig. 2), CFU counts decreased progressively with both concentration and duration of gentamicin exposure. Bacterial counts did not fall below the LLOD systematically, even at 2000 mg L^−1^. The addition of rifampin (3.3 mg L^−1^) did not improve the proportion of samples with bacterial counts below the LLOD. CFU counts were comparable or even slightly higher at several timepoints compared with gentamicin alone. This finding was consistent across the three independent replicates ( n=18 for gentamicin with rifampin; n=6 for gentamicin alone).

For *intermittent twice-daily exposure* (Fig. 3), a reduction in CFU was again observed over time, particularly at higher gentamicin concentrations (250 and 2000 mg L^−1^). Nevertheless, viable bacteria persisted throughout the 28 d experiment. Rifampin addition modestly accelerated bacterial reduction at early timepoints, but final CFU levels remained comparable to those of gentamicin monotherapy.

For the *burst-release regimen* (Fig. 4), CFU counts dropped markedly during the initial days of high-concentration exposure but rebounded after concentrations fell below approximately 20 mg L^−1^. No samples reached bacterial counts below the LLOD by day 14, even with concurrent rifampin.

The number of pegs with bacterial counts below the LLOD for each condition is summarised in Table 2, while detailed CFU data are provided in Tables S1–S3 in the Supplement, corresponding to the continuous, intermittent and burst-release regimens, respectively.

**Table 2:**
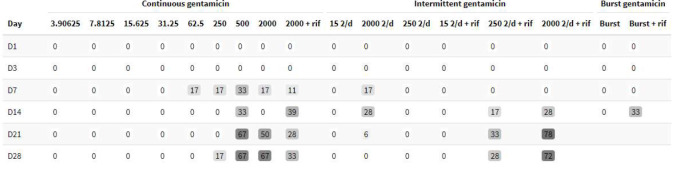
Percentage of pegs with bacterial counts below the lower limit of detection (LLOD) at each timepoint. The effective LLOD was 10 CFUs per peg.

**Figure 5:**
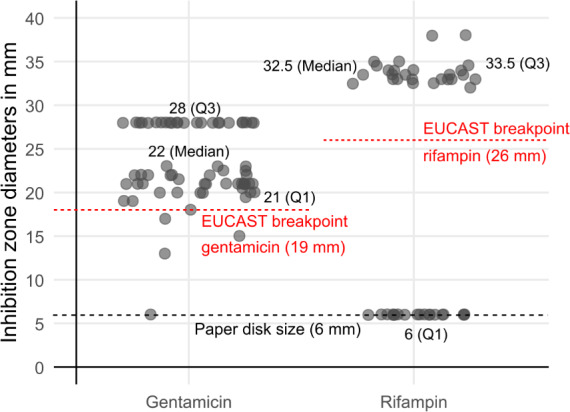
Inhibition zone diameters of selected colonies surviving antibiotic exposure, tested for gentamicin and rifampin according to EUCAST guidelines. Each point represents an individual measurement. The dashed line at 6 mm marks the paper-disc diameter. While most gentamicin inhibition zone diameters remained above the susceptibility breakpoint ( ≥18 mm), a subset of post-exposure isolates showed rifampin inhibition zone diameters below the EUCAST susceptibility breakpoint ( ≤26 mm).

Across all regimens, inhibition-zone diameters for gentamicin remained predominantly within the susceptible range (median 22 mm; range 6–28). Rifampin inhibition zones also remained largely within the susceptible range, although isolated colonies displayed resistance (zone diameter 6 mm). Individual results for both antibiotics are illustrated in Fig. 5. Resistances were observed in isolated samples throughout all concentrations and all exposure regimens. Small numbers do not allow further statistical analysis.

## 4 Discussion

Biofilm formation is one of the principal drivers of persistence and relapse in ODRI (Costerton et al., 1987; Gristina, 1987; Bjarnsholt et al., 2013a; Harms et al., 2016). It offers microorganisms increased tolerance to antibiotics and to the action of the immune system (Brauner et al., 2016; Olsen, 2015), and allows dormant cells to survive, offering possibilities for relapse in improved conditions or if antibiotic resistance develops (Harms et al., 2016). Therefore, biofilm eradication is a key element for treatment success in ODRI. This study tested if biofilm may be eradicated by gentamicin, one of the most commonly used antibiotics in local therapy, as may be observed for other antibiotics, particularly vancomycin (Baeza et al., 2019; Post et al., 2017).

However, gentamicin failed to eradicate 5 d old *S. aureus* biofilm in this study, regardless of administration scheme or concentration tested, even at concentrations exceeding cellular toxicity thresholds and with the addition of rifampin (Ehanire et al., 2007; Chang et al., 2006; Antoci et al., 2007; Kallala et al., 2012; Ince et al., 2007; Rathbone et al., 2011; Renz et al., 2021; Beldman et al., 2021). As cellular toxicity is time dependent, with thresholds decreasing with increasing duration of exposure (Rathbone et al., 2011; Wiesli et al., 2021), no single value may be indicated. The duration of exposure tested also exceeded by far release from carriers made of antibiotic bone cement (Anagnostakos et al., 2009). The results of this study question the routine use of aminoglycosides in the treatment of ODRI, at least when applied without combination with other antibiotics or implant removal. Furthermore, aminoglycosides have no significant activity against intracellular persisters (Sanchez et al., 1986; Easmon, 1979), another of the main persistence modes of bacteria in orthopaedic infections (Kalinka et al., 2014; Sendi and Proctor, 2009; Tuchscherr et al., 2010; Harms et al., 2016). Our findings help to explain the variable cure rates reported for gentamicin-loaded carriers in chronic osteomyelitis (McNally et al., 2016, 2022) or periprosthetic joint infection (Reinisch et al., 2022; Sigmund et al., 2024; Flierl et al., 2017; Tarity et al., 2022; Gramlich et al., 2020): high local concentrations alone of this antibiotic do not sterilise mature biofilm.

Gentamicin was selected because aminoglycosides remain the most frequently used antibiotics for local application in orthopaedic and trauma surgery (Antoci et al., 2007; Kallala et al., 2012; Kluin et al., 2013; Hake et al., 2015; Iarikov et al., 2012). There is no significant difference between gentamicin and tobramycin regarding dosing and antimicrobial activity (Krause et al., 2016). Therefore, our observations may be extrapolated to tobramycin. The failure observed in this study may be explained by the mechanism of action of aminoglycosides. The main mode of action of aminoglycosides is by disrupting protein synthesis (Krause et al., 2016; Taber et al., 1987). This requires intracellular penetration of the drug by an active, oxygen-dependent transport with exchange against a proton (Taber et al., 1987; Krause et al., 2016). The low pH, limited oxygen availability and reduced metabolic rates within mature biofilm reduce both uptake and target engagement (Krause et al., 2016). Although a part of the bactericidal action of aminoglycosides is by direct damage to the bacterial cell wall (Kadurugamuwa et al., 1993; Krause et al., 2016), this may, however, not be sufficient for a decisive effect under biofilm conditions. While local antibiotics are frequently employed in ODRI treatment, their selection has historically been guided by precedent use rather than robust evidence-based protocols. A deeper understanding of how antibiotics contribute to ODRI treatment is necessary to refine and improve these protocols, ensuring more effective and targeted therapeutic strategies. At least for Gram-positive bacteria in a biofilm, vancomycin may be a better choice (Baeza et al., 2019; Post et al., 2017; Wahl et al., 2017). Ceftriaxone in combination with calcium sulfate (CaSO_4_) may offer further interesting options as the carrier provides a particularly steady and prolonged local release (Wiesli et al., 2021, 2022; Wahl et al., 2018).

The intermittent exposure to gentamicin 2 h twice daily was chosen to mirror the serum peak-and-trough pattern of traditional q12 hours systemic gentamicin therapy, matching presumed optimal pharmacodynamics (Hanberger et al., 2013). The culture media were exchanged after the 2 h for antibiotic-free media. The burst exposure pattern had been chosen to simulate release from a local carrier as used typically in orthopaedic surgery (Anagnostakos et al., 2009), as well as the declining concentrations observed in a large animal infection model (Boot et al., 2021). Rifampin was added at a concentration of 3.3 mg L^−1^, the mean peri-implant extracellular-fluid concentration observed in a sheep pharmacokinetic study (Boot et al., 2021). While such a concentration may be maintained even 12 h after administration of a single dose of rifampin (Acocella, 1978), it already reaches a concentration threshold for cellular toxicity (Isefuku et al., 2001). Such thresholds are, however, difficult to define precisely, as both concentration and duration of exposure determine cellular toxicity (Wiesli et al., 2021; Rathbone et al., 2011). So far, no evidence allows for the definition of better targets for biofilm treatment, but higher concentrations may be expected to be associated with more local complications. However, some bacteria have even higher minimal inhibitory concentrations for rifampin (Lepe et al., 2012). As rifampin is a concentration-dependent antibacterial drug (Gumbo et al., 2007), the continuous administration chosen for this study, reflecting best usual systemic administration, may not have exploited the full effect of this antibiotic.

*S. aureus* was selected as the test organism because staphylococci account for the majority of ODRI and osteomyelitis (Zuluaga et al., 2006; Schwotzer et al., 2014; Holleyman et al., 2016; Arciola et al., 2005). Biofilm was allowed to mature for 5 d before antibiotic exposure (Fig. 1), and 1 % plasma was added to the culture media to ensure a fully developed extracellular matrix, as typically observed in vivo (Hartmann et al., 2025; Isguven et al., 2022). The addition of plasma to TSB was chosen to introduce selected host-associated components and thereby improve the translational relevance of the in vitro biofilm model compared with protein-free media (Isguven et al., 2022). This approach avoids overestimating antibiotic efficacy, since immature biofilms are more susceptible to antimicrobial stress. Conversely, eradication is expected to be even more challenging under true in vivo conditions, where shear stress, nutrient limitation and host-derived signals further increase tolerance (Post et al., 2017; Hartmann et al., 2025). For instance, vancomycin eradicated *S. aureus* biofilm in vitro under static conditions but not if the wells were agitated, emphasising the impact of mechanical stresses (Post et al., 2017). It is recognised that plasma proteins may influence apparent antibiotic activity through reversible binding or partial inactivation, particularly for antibiotics with high plasma protein binding (Mouton et al., 2008). The influence of proteins on antibiotic activity represents an inherent and widely acknowledged limitation of in vitro biofilm models incorporating host components (Isguven et al., 2022). This effect may be neglected, as wound fluid has a protein content more than 50 times higher and, as protein binding, would be saturated at the concentrations tested (James et al., 2000; Trengove et al., 1996; Bodendorf et al., 2007). Furthermore, gentamicin is a small hydrophilic aminoglycoside with low plasma protein binding, and its antibacterial activity is generally preserved in protein-containing environments (Mouton et al., 2008; Boot et al., 2021). In contrast, rifampin exhibits high but reversible plasma protein binding, which may reduce the freely available fraction under in vitro conditions (Niemi et al., 2003). These effects are concentration dependent, and, at the high local antibiotic concentrations applied in the present model, pharmacologically relevant free drug levels are expected to remain present (Boot et al., 2021; Isguven et al., 2022; Wiesli et al., 2022). As all experimental conditions were performed using identical media composition, any plasma-related effects would apply uniformly across treatment regimens and are therefore unlikely to explain the observed lack of biofilm eradication.

Disc-diffusion testing of post-exposure isolates showed no regular emergence of gentamicin resistance, while rifampin resistance was observed in a subset of recovered colonies. Alongside persistent CFU counts, this supports phenotypic biofilm tolerance as the primary barrier. Even where rifampin susceptibility was retained, biofilm persisted, highlighting the fact that higher gentamicin exposure is insufficient for eradication. The persistent biofilm, therefore, reflects phenotypic tolerance rather than the selection of genetically resistant clones, a phenomenon well documented for *S. aureus* biofilm (Lamret et al., 2020; Brauner et al., 2016; Conlon et al., 2016). Although high local peaks can suppress resistance emergence when subinhibitory tails are avoided (Awad et al., 2013; Mouton et al., 2008; Gullberg et al., 2011), our data show that simply increasing gentamicin concentration is insufficient for biofilm eradication. Because only colonies from culture-positive pegs were tested, these results document the occurrence rather than the prevalence of resistance.

Although biofilm formation is central to ODRI pathology, treatment success also depends on factors such as microbial spectrum, tissue penetration and intracellular persistence, making antibiotic selection complex. This may well be illustrated by the results of a relatively large study on the treatment of osteomyelitis and fracture-related infection, where the addition of vancomycin to aminoglycoside-loaded CaSO_4_ carrier material did not significantly improve outcomes (Unsworth et al., 2024). This underscores the fact that factors beyond biofilm formation play a significant role, at least in bone infections. Yet a broad spectrum alone may not decide success, as in a large randomised trial on diabetic foot infection, linezolid – active only against Gram-positive bacteria – matched the outcome of aminopenicillins in combination with a penicillinase inhibitor, which has a much broader antibacterial spectrum (Lipsky et al., 2004). Systemic as well as local antibiotic use is further constrained by tolerance, hypersensitivity and toxicity. Beyond biofilm (Costerton et al., 1987; Gristina, 1987; Høiby et al., 2010; Bjarnsholt et al., 2013a; Olsen, 2015; Harms et al., 2016; Hartmann et al., 2025), polymicrobial flora, intracellular persisters and small-colony variants (Sendi and Proctor, 2009; Kalinka et al., 2014; Tuchscherr et al., 2010; Harms et al., 2016; Hamad et al., 2022), and limited tissue penetration (Mouton et al., 2008) all undermine success and should all be addressed when selecting antimicrobial agents. While local administration may be favoured by certain pharmacodynamic arguments, it remains limited by cellular toxicity (Rathbone et al., 2011; Wiesli et al., 2021) as well as by drug stability (Samara et al., 2017). Further research is warranted, but as gentamicin failed to eradicate biofilm in these experiments, the single use of aminoglycosides in the local treatment of ODRI has to be questioned.

## Supplement

10.5194/jbji-11-65-2026-supplement The supplement related to this article is available online at https://doi.org/10.5194/jbji-11-65-2026-supplement.

## Data Availability

The data supporting the findings of this study are available from the corresponding author upon reasonable request.
