# Supplementary material for: Gentamicin fails to eradicate Staphylococcus aureus biofilm in vitro, even in combination with rifampin
Source: J Bone Jt Infect. 2026 Feb 2;11(1):65–76. doi: 10.5194/jbji-11-65-2026 (PMC12891982; doi:10.5194/jbji-11-65-2026)
Supplement: The supplement related to this article is available online at https://doi.org/10.5194/jbji-11-65-2026-supplement. [file jbji-11-65-2026-supplement.pdf]

Supplement of J. Bone Joint Infect., 11, 65–76, 2026
https://doi.org/10.5194/jbji-11-65-2026-supplement
© Author(s) 2026. CC BY 4.0 License.

JBJI
Journal of Bone and Joint Infection
Open Access

Supplement of

Gentamicin fails to eradicate *Staphylococcus aureus* biofilm in vitro, even in combination with rifampin

Willemijn Boot et al.

Correspondence to: Peter Wahl (peter.wahl@faculty.unibe.ch)

The copyright of individual parts of the supplement might differ from the article licence.

Table S1

Gentamicin Concentration (mg/L)	Day 1 Median (Min - Max)	Day 3 Median (Min - Max)	Day 7 Median (Min - Max)	Day 14 Median (Min - Max)	Day 21 Median (Min - Max)	Day 28 Median (Min - Max)
0	9.4e+07 (6.0e+07 - 3.3e+08)	2.9e+08 (2.1e+08 - 4.5e+08)	2.1e+08 (8.0e+07 - 9.3e+08)	6.5e+08 (4.4e+08 - 1.0e+09)	4.2e+08 (3.1e+08 - 8.8e+08)	3.1e+08 (2.3e+08 - 7.1e+08)
0.98	1.4e+08 (1.1e+08 - 2.8e+08)	1.9e+08 (7.2e+07 - 4.0e+08)	3.6e+08 (2.4e+08 - 4.7e+08)	1.1e+09 (4.7e+08 - 1.3e+09)	5.4e+08 (3.4e+08 - 9.5e+08)	2.8e+08 (2.2e+08 - 5.9e+08)
1.95	1.0e+08 (5.6e+07 - 2.4e+08)	7.2e+07 (4.3e+07 - 2.7e+08)	1.3e+07 (1.0e+06 - 1.7e+08)	3.1e+08 (8.3e+07 - 1.0e+09)	2.3e+08 (9.5e+07 - 4.0e+08)	2.6e+08 (1.1e+08 - 4.2e+08)
3.91	4.9e+07 (3.5e+07 - 2.4e+08)	6.0e+07 (5.3e+06 - 2.4e+08)	1.3e+07 (5.0e+05 - 8.6e+07)	2.0e+08 (9.7e+07 - 3.6e+08)	2.1e+08 (1.0e+08 - 4.2e+08)	2.1e+08 (1.8e+08 - 3.2e+08)
7.81	3.8e+07 (2.3e+07 - 2.5e+08)	7.2e+07 (8.3e+06 - 2.4e+08)	3.7e+06 (1.0e+06 - 5.6e+07)	3.1e+08 (1.2e+08 - 6.7e+08)	1.5e+08 (3.4e+07 - 2.9e+08)	2.0e+08 (9.2e+07 - 6.1e+08)
15.63	4.0e+07 (1.2e+07 - 1.0e+08)	3.3e+07 (6.5e+06 - 1.3e+08)	1.2e+06 (4.0e+05 - 7.1e+07)	1.1e+08 (5.4e+07 - 1.9e+08)	7.3e+07 (4.0e+07 - 1.6e+08)	1.7e+08 (7.1e+07 - 6.8e+08)
31.25	4.7e+07 (1.2e+07 - 2.6e+08)	7.3e+06 (5.8e+06 - 1.7e+08)	5.1e+05 (2.1e+05 - 1.2e+07)	8.7e+07 (7.3e+07 - 1.2e+08)	6.7e+07 (3.6e+06 - 2.5e+08)	1.6e+08 (6.4e+07 - 3.8e+08)
62.5	3.7e+07 (6.8e+06 - 9.7e+07)	1.5e+07 (3.8e+06 - 2.2e+08)	4.6e+05 (0.0e+00 - 1.6e+07)	3.1e+07 (6.1e+06 - 7.5e+07)	1.0e+08 (2.6e+07 - 1.7e+08)	1.8e+08 (1.2e+08 - 3.1e+08)
125	1.6e+07 (3.1e+06 - 1.1e+08)	1.7e+07 (4.9e+06 - 1.5e+08)	4.6e+05 (0.0e+00 - 1.6e+07)	4.0e+07 (9.2e+06 - 7.0e+07)	1.8e+07 (9.7e+04 - 6.3e+07)	9.0e+07 (8.0e+05 - 3.1e+08)
250	1.3e+07 (1.0e+06 - 5.8e+08)	1.2e+07 (4.4e+04 - 7.0e+07)	4.4e+05 (0.0e+00 - 1.6e+07)	6.4e+06 (2.0e+01 - 1.6e+07)	7.4e+06 (3.6e+02 - 2.1e+07)	1.2e+04 (0.0e+00 - 3.0e+06)

Gentamicin Concentration (mg/L)	Day 1 Median (Min - Max)	Day 3 Median (Min - Max)	Day 7 Median (Min - Max)	Day 14 Median (Min - Max)	Day 21 Median (Min - Max)	Day 28 Median (Min - Max)
500	5.1e+06 (2.0e+05 - 6.3e+07)	7.4e+06 (1.4e+05 - 5.7e+07)	5.4e+05 (0.0e+00 - 1.6e+07)	4.2e+02 (0.0e+00 - 1.6e+05)	0.0e+00 (0.0e+00 - 1.5e+04)	0.0e+00 (0.0e+00 - 9.1e+02)
1000	1.2e+07 (7.0e+05 - 5.4e+07)	1.4e+06 (1.6e+05 - 4.5e+07)	0.0e+00 (0.0e+00 - 4.7e+05)	2.6e+02 (0.0e+00 - 1.3e+05)	0.0e+00 (0.0e+00 - 1.0e+01)	0.0e+00 (0.0e+00 - 6.0e+01)
2000	6.0e+06 (1.6e+06 - 2.3e+07)	3.5e+05 (4.1e+04 - 1.7e+06)	3.5e+01 (0.0e+00 - 5.7e+04)	1.0e+02 (1.0e+01 - 1.1e+05)	1.0e+01 (0.0e+00 - 9.0e+02)	0.0e+00 (0.0e+00 - 6.0e+01)
2000 + rifampin 3.3 mg/L	1.3e+08 (5.0e+07 - 6.6e+08)	2.3e+06 (3.0e+05 - 1.6e+07)	7.4e+04 (0.0e+00 - 5.5e+06)	8.0e+01 (0.0e+00 - 6.3e+06)	3.4e+02 (0.0e+00 - 6.5e+04)	1.2e+02 (0.0e+00 - 2.3e+04)

Table S1 – Median and range of colony forming units for the continuous exposure to gentamicin, including with addition of rifampin at 3.3 mg/L to the maximum concentration of gentamicin of 2'000 mg/L.

Table S2

Gentamicin Concentration (mg/L)	Day 1 Median (Min - Max)	Day 3 Median (Min - Max)	Day 7 Median (Min - Max)	Day 14 Median (Min - Max)	Day 21 Median (Min - Max)	Day 28 Median (Min - Max)
0	3.8e+08 (9.6e+07-9.8e+08)	2.5e+08 (6.4e+07-5.9e+08)	3.4e+08 (1.6e+08-5.7e+08)	4.0e+08 (2.7e+08-6.5e+08)	3.0e+08 (1.0e+08-7.0e+08)	6.4e+08 (2.7e+08-1.3e+09)
15	2.4e+08 (7.5e+07-1.1e+09)	2.0e+08 (6.1e+07-9.2e+08)	2.9e+08 (1.2e+08-7.6e+08)	3.3e+08 (1.3e+08-8.0e+08)	2.3e+08 (1.3e+08-7.0e+08)	3.1e+08 (1.7e+08-8.8e+08)
250	2.4e+08 (4.8e+07-9.6e+08)	2.0e+07 (6.1e+06-1.8e+08)	1.8e+07 (1.1e+04-1.4e+08)	9.4e+07 (1.7e+07-2.5e+08)	1.3e+08 (1.6e+07-3.8e+08)	4.2e+08 (1.9e+08-1.2e+09)
2000	1.6e+08 (1.8e+07-6.4e+08)	3.1e+06 (2.3e+05-2.9e+07)	2.7e+03 (0.0e+00-5.0e+06)	8.7e+04 (0.0e+00-9.0e+06)	1.2e+04 (0.0e+00-3.8e+07)	6.0e+05 (2.6e+02-1.4e+08)
0 + rifampin	3.8e+08 (1.0e+08 - 8.6e+08)	3.0e+08 (1.0e+08-8.6e+08)	5.5e+08 (8.0e+07-2.1e+09)	n/a	n/a	6.2e+08 (3.0e+08-8.4e+08)
15 + rifampin	3.6e+08 (1.4e+08-8.6e+08)	1.4e+08 (5.1e+07-3.5e+08)	5.6e+07 (1.1e+06-2.5e+08)	3.0e+08 (1.2e+07-7.8e+08)	6.6e+08 (1.2e+08-1.2e+09)	7.4e+08 (4.6e+08-2.5e+09)
250 + rifampin	3.1e+08 (1.1e+08-6.6e+08)	1.0e+08 (8.0e+06-3.0e+08)	3.5e+06 (4.1e+05-6.6e+07)	8.6e+02 (0.0e+00-6.2e+06)	6.5e+02 (0.0e+00-7.3e+06)	4.4e+02 (0.0e+00-6.8e+08)
2000 + rifampin	2.3e+08 (1.1e+08-3.1e+09)	5.7e+07 (1.2e+07-2.5e+08)	1.8e+06 (3.2e+02-1.6e+07)	2.0e+02 (0.0e+00-6.4e+06)	0.0e+00 (0.0e+00-2.8e+03)	0.0e+00 (0.0e+00-3.6e+07)

Table S2 – Median and range of colony forming units for the exposure to gentamicin twice daily for two hours, including with addition of rifampin at 3.3 mg/L. “n/a” indicates data not available.

Table S3

Gentamicin Concentration (mg/L)	Day 1 Median (Min - Max)	Day 3 Median (Min - Max)	Day 7 Median (Min - Max)	Day 14 Median (Min - Max)
0	2.0e+08 (1.0e+08 - 9.5e+08)	4.1e+08 (2.1e+08 - 2.1e+09)	2.6e+08 (3.7e+07 - 6.7e+08)	2.6e+08 (1.0e+08 - 5.4e+08)
Burst challenge	2.6e+07 (1.3e+07 - 5.0e+07)	2.5e+07 (1.3e+07 - 8.0e+07)	1.5e+07 (2.1e+06 - 4.5e+07)	3.0e+08 (8.8e+07 - 9.9e+08)
Burst challenge + rifampin 3.3 mg/L	1.2e+08 (2.4e+07 - 2.8e+08)	5.3e+07 (2.5e+07 - 1.7e+08)	2.7e+06 (9.3e+05 - 1.9e+07)	1.1e+03 (0.0e+00 - 2.7e+05)

Table S3 – Median and range of colony forming units for the burst exposure to gentamicin, simulating release from a carrier material, including with addition of rifampin at 3.3 mg/L.
